# Supplementary material for: Polyoxovanadate-Based Cyclomatrix Polyphosphazene Microspheres as Efficient Heterogeneous Catalysts for the Selective Oxidation and Desulfurization of Sulfides
Source: Molecules. 2022 Dec 5;27(23):8560. doi: 10.3390/molecules27238560 (PMC9738953; doi:10.3390/molecules27238560)
Supplement: Supplementary file 1 [file molecules-27-08560-s001.zip › molecules-2038994-supplementary.pdf]

Supporting Information

Polyoxovanadate-Based Cyclomatrix Polyphosphazene Microspheres as Efficient Heterogeneous Catalysts for the Selective Oxidation and Desulfurization of Sulfides

Yinghui Hu [1,†], Diping Huang [1,†], Jing Yan [1,*], Zhiliang Miao [1], Lize Yu [2], Ningjing Cai [2], Quanhai Fang [1], Qiuyu Zhang [1] and Yi Yan [1,*]

1 Department of Chemistry, School of Chemistry and Chemical Engineering, Key Laboratory of Special Functional and Smart Polymer Materials of Ministry of Industry and Information Technology, Northwestern Polytechnical University, Xi'an 710129, China
2 Queen Mary University of London Engineering School, Northwestern Polytechnical University, Xi'an 710129, China
* Correspondence: yanjing@nwpu.edu.cn (J.Y.); yanyi@nwpu.edu.cn (Y.Y.)
† These authors contributed equally to this work.

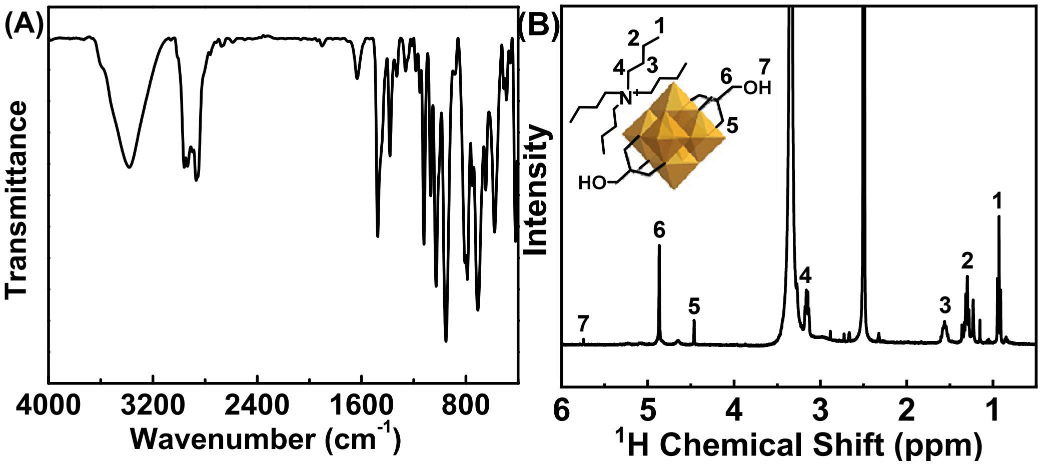

Figure S1. FT-IR spectrum (A) and ^{1}H NMR spectrum (B) of V_6O_{13}-OH in DMSO-*d*$_6$.

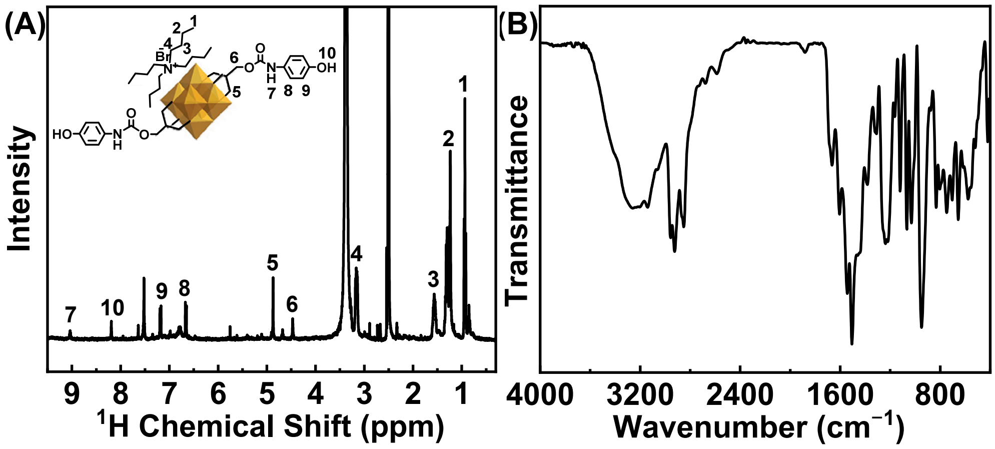

Figure S2. ^{1}H NMR spectrum (A) in DMSO-*d*$_6$ and FT-IR spectrum (B) of V_6O_{13}-PhOH.

TGA of HCCP-V

The calculation of the proportion of V_6 in HCCP-V: The mass of the sample put into the crucible is 6.032 mg, and the mass residual rate is 42.52% at 900 °C. Assuming that the final residual P_2O_5 and V_2O_5 of the sample are x mmol and y mmol respectively, the following equation can be obtained:

$$142\,x + 182\,y = 2.565$$

$$2 * 135x/3 + 1532y/3 = 6.032$$

Solving for x= 0.003777, y= 0.01115

P_3N_3 is 0.002518 mmol, V_6O_{13}-PhOH is 0.003717 mmol

P_3N_3: V_6O_{13}-PhOH= 1: 1.476

Then the weight percentage of V_6 in **HCCP-V** is 18.85%.

SEM of HCCP-V

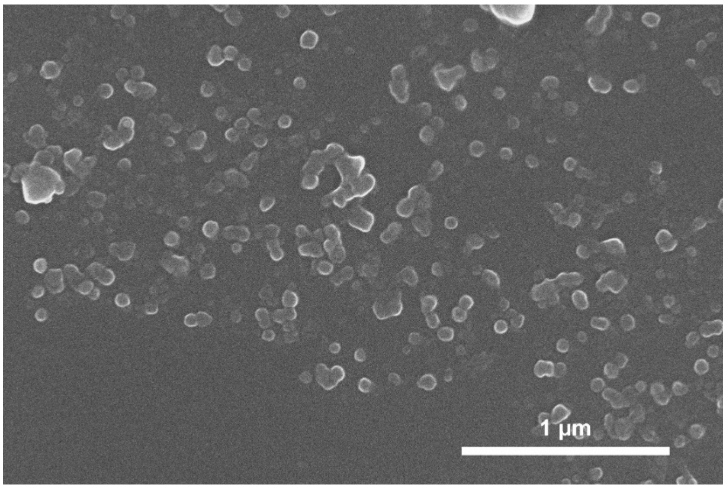

Figure S3. SEM image of **HCCP-V** dispersed in EtOH.

The HPLC standard curve of MPSO

Prepare MPSO solutions of different concentrations, 0.5 mM, 1 mM, 2 mM, 3 mM, 4 mM and 5 mM, using high performance liquid chromatography under the same mobile phase conditions as the test environment (acetonitrile:H_2O = 7:3, 10 μL) to give the standard curve.

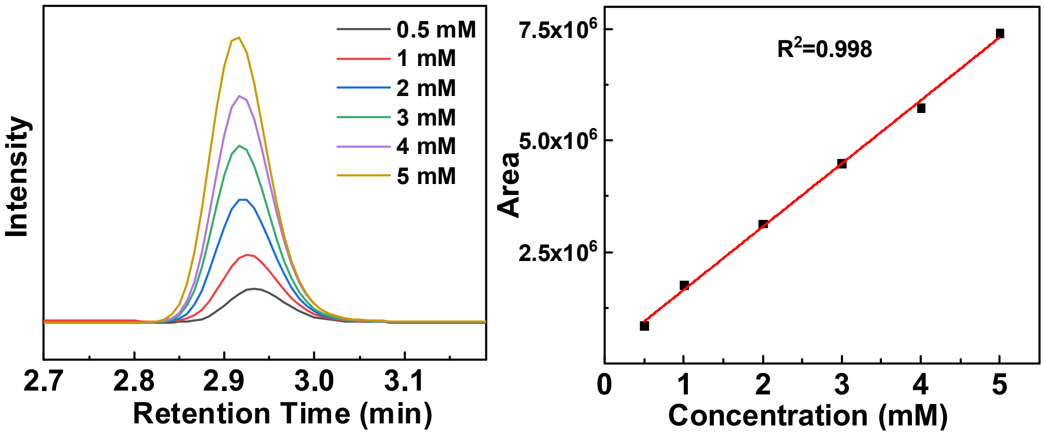

Figure S4. HPLC and standard curve of MPSO.

The blank and controlled experiment of MPS oxidation

The reaction temperature is 40 ℃ and the ratio is [MPS]:[H_2O_2]:[catalyst]= 1:1.2:1/400. Within 180 min of the reaction time, the peak of MPS remained unchanged, and the peaks of MPSO and MPSO2 did not appear at 2.7 min and 3.3 min, indicating the catalytic oxidation process of MPS was very slow when no catalyst was added, or HCCP-BPS was used as the catalyst. Naphthalene was added as internal standard to eliminate errors, and its retention time was 10.2 min.

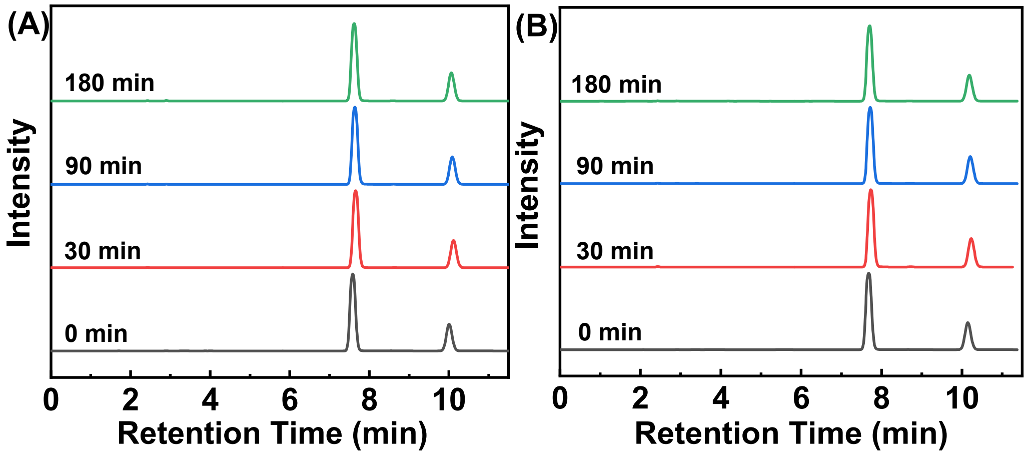

Figure S5. Catalytic oxidation of MPS with H_2O_2 as oxidant: (A) no catalyst; (B) **HCCP-BPS**.

^{1}H NMR spectra of MPS oxidation product

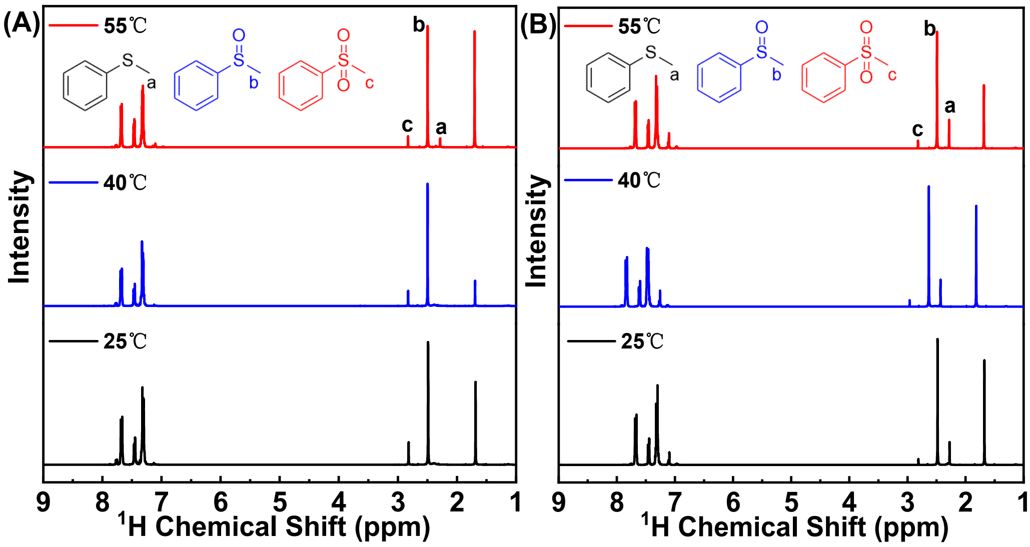

Figure S6. ^{1}H NMR spectra in $CDCl_3$ for the product of the catalytic oxidation of

MPS with different ratio of **HCCP-V** at different temperature (25 °C, 40 °C, and 55 °C): (A) 1.2 eq, (B) 1 eq.

According to the ^{1}H NMR spectra, it can be seen that when the oxidant equivalent is 1.2 and the reaction temperature is 25 °C, only the peaks of MPSO and MPSO2 appear at 2.49 ppm and 2.83 ppm, indicating that anisole sulfide completely oxidized. At 40 °C, the peaks of MPSO and MPSO2 appear at 2.49 ppm and 2.83 ppm, and the peak of MPSO2 is weakened, indicating the selectivity improved. At 55 °C, the peaks of MPS, MPSO and MPSO2 appear at 2.29 ppm, 2.49 ppm and 2.83 ppm, indicating MPS is not fully oxidized. At 25 °C, 40 °C and 55 °C with oxidant dosage of 1 eq, the signals of MPS, MPSO and MPSO2 appear at 2.29 ppm, 2.49 ppm and 2.83 ppm, indicating that the oxidant dosage added is not enough. Therefore, it is proved that the optimal reaction conditions for the catalytic oxidation of MPS are oxidant 1.2 eq at 40 °C.

The HPLC standard curve of DBTSO2

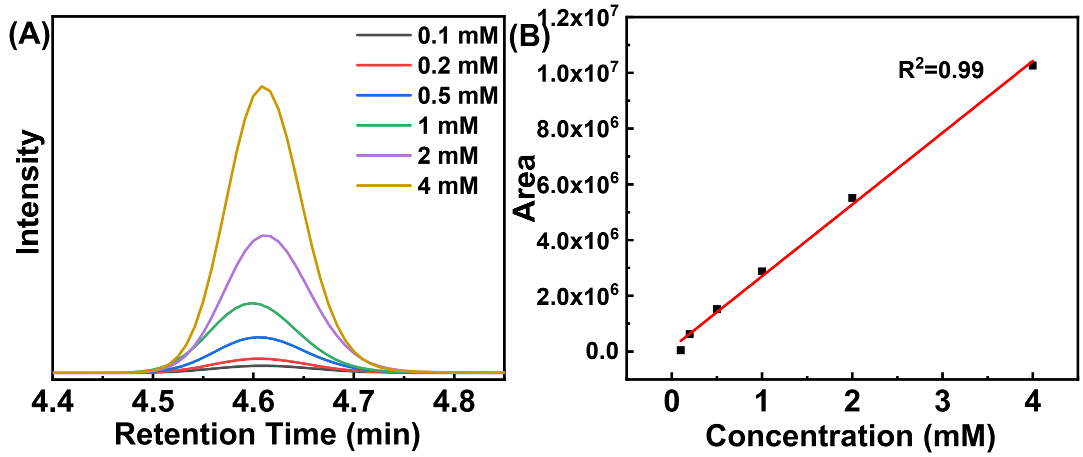

Figure S7. HPLC and standard curve of DBTSO2.

The recycle experiment of MPS oxidation

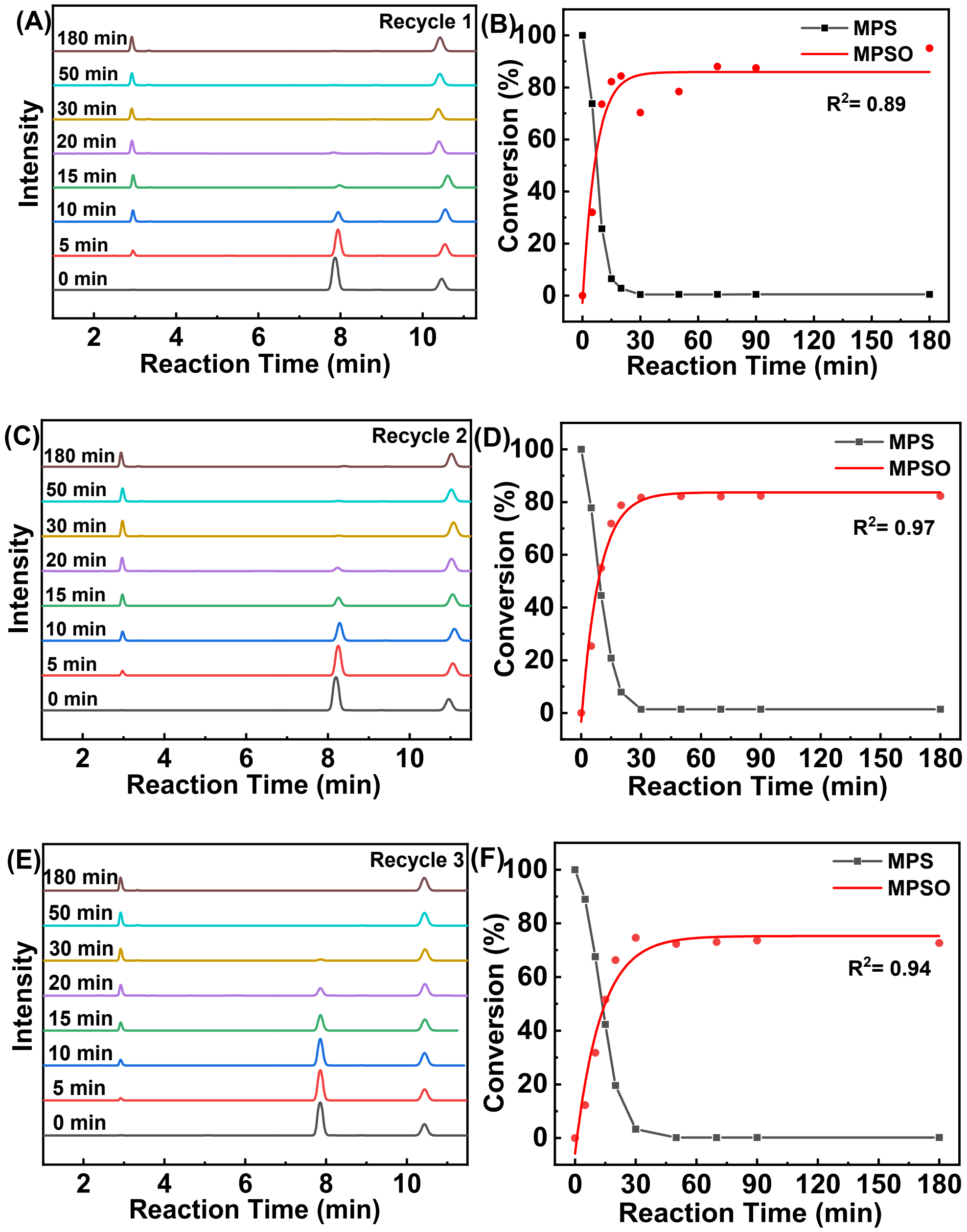

Figure S8. HPLC traces and conversion curve of MPS oxidation during recycle experiment.

Comparison with other catalysts

Table S1. Comparison with other POM-based catalysts for catalytic oxidation of different sulfides.

Catalyst	Substrate	Solvent	Temperature (°C)	$[H_2O_2]$:[Catalyst]	Time (min)	Conv. (%)	Sel. (%)[a]	Ref.
TBA-polyV_6	CEES	H_2O_2/EtOH	25 °C	1.2:1/20	40	100	100.0	[1]
V–Co-MOF	CEES	H_2O_2/EtOH	25 °C	1.2:1/10	10	100	100.0	[2]
$[As^{III}Mo_6O_{21}(PABA)_3]_2$	CEES	H_2O_2/CH_3CN	25 °C	1.2:1/100	12	98.9	98.0	[3]
$[AsMo_6O_{21}(Ala)(PHBA)_2]$	CEES	H_2O_2/EtOH	25 °C	1:3/200	5	98.5	>99.9	[4]
**HCCP-V**	CEES	H_2O_2/CH_3CN	25 °C	1.2:1/400	60	98.2	>99.9	This work
$[Co_2Mo_{10}H_4O_{38}]$	MPS	TBHP/CH_3CN	40 °C	1:3/200	240	99	100.0	[5]
$[V_4O_{12}]$	MPS	H_2O_2/EtOH	45 °C	1.2:7/200	240	98.6	98.7	[6]
$[P^{III}Mo_6O_{21}(PABA)_3]_2$	MPS	H_2O_2/EtOH	25 °C	1.2:1/100	20	99.5	98.0	[7]
$[Co(HDTBA)V_2O_6]$	MPS	TBHP/CH_3OH	50 °C	1.5:3/500	15	100	99.0	[8]
**HCCP-V**	MPS	CH_3CN	40°C	1.2:1/400	50	99.6	>99.9	This work
$[HPMo][HTAC]_2/SiO_2$	DBT	*n*-octane	70 °C	1.12:1	300	91	95.0	[9]
$[Omim]_3PMo_{12}O_{40}$/KIT-6	DBT	*n*-octane	60 °C	5:1	80	100	97.8	[10]
P[Vim]POM/GO	DBT	*n*-octane	60 °C	9:1	60	100	>98.0	[11]
V19/Al	DBT	diesel/CH_3CN	60 °C	4:1	30	98.6	>96.0	[12]
**HCCP-V**	DBT	CH_3CN	70 °C	8:1/100	50	92.2	98.9	This work

[a]Selectivity to corresponding sulfoxide (CEESO, MPSO, and DBTSO2).

Reference:

1. Tian, H.; Zhang, Z.; Dang, T.; Liu, S.; Lu, Y.; Liu, S., Hollow lindqvist-like-shaped {V_6} cluster-based metal-organic framework for the highly efficient detoxification of mustard gas simulant. *Inorg Chem* **2021,** 60, 840–845.
2. Tian, H.; Zhang, Z.; Liu, S.; Dang, T.; Li, X.; Lu, Y.; Liu, S., A novel polyoxovanadate-based Co-MOF: Highly efficient and selective oxidation of a mustard gas simulant by two-site synergetic catalysis. *J Mater Chem A* **2020,** 8, 12398–12405.
3. Hou, Y.; An, H.; Chang, S.; Zhang, J., Versatile catalysts constructed from hybrid polyoxomolybdates for simultaneously detoxifying sulfur mustard and organophosphate simulants. *Catal Sci Technol* **2019,** 9, 2445–2455.
4. Hou, Y.; An, H.; Zhang, Y.; Hu, T.; Yang, W.; Chang, S., Rapid destruction of two types of chemical warfare agent simulants by hybrid polyoxomolybdates modified by carboxylic acid ligands. *ACS Catal* **2018,** 8, 6062–6069.
5. An, H.; Hou, Y.; Wang, L.; Zhang, Y.; Yang, W.; Chang, S., Evans-showell-type polyoxometalates constructing high-dimensional inorganic-organic hybrid compounds with copper-organic coordination complexes: Synthesis and oxidation catalysis. *Inorg Chem* **2017,** 56, 11619–11632.
6. Li, J.; Wei, C.; Guo, D.; Wang, C.; Han, Y.; He, G.; Zhang, J.; Huang, X.; Hu, C., Inorganic-organic hybrid polyoxovanadates based on $[V_4O_{12}]^{4-}$ or $[VO_3]_2{}^{2-}$ clusters: Controllable synthesis, crystal structures and catalytic properties in selective oxidation of sulfides. *Dalton Trans* **2020,** 49, 14148–14157.
7. An, H.; Hou, Y.; Chang, S.; Zhang, J.; Zhu, Q., Highly efficient oxidation of various thioethers catalyzed by organic ligand-modified polyoxomolybdates. *Inorg Chem Front* **2020,** 7, 169–176.
8. Wang, X.; Zhang, T.; Li, Y.; Lin, J.; Li, H.; Wang, X., In situ ligand-transformation-involved synthesis of inorganic-organic hybrid polyoxovanadates as efficient heterogeneous catalysts for the selective oxidation of sulfides. *Inorg Chem* **2020,** 59, 17583–17590.
9. Li, Q.; Wang, G.; Qiu, J.; Wang, N.; Zhang, Q.; Lei, Q.; Hu, Y. L.; Zhang, Y., Catalytic oxidative desulfurization of model fuel using $[HPMo][HTAC]_2/SiO_2$ as a amphiphilic catalyst. *Adv Mater Res* **2011,** 396–398, 827–832.
10. Ding, Y.; Wang, J.; Liao, M.; Li, J.; Zhang, L.; Guo, J.; Wu, H., Deep oxidative desulfurization of dibenzothiophene by novel POM-based IL immobilized on well-ordered KIT-6. *Chem Eng J* **2021,** 418, 129470.
11. Gao, Y.; Cheng, L.; Gao, R.; Hu, G.; Zhao, J., Deep desulfurization of fuels using supported ionic liquid-polyoxometalate hybrid as catalyst: A comparison of different types of ionic liquids. *J Hazard Mater* **2021,** 401, 123267.
12. Cedeño, L.; Gomez, H.; Fraustro, A.; Guerra, H.; Cuevas, R., Oxidative desulfurization of synthetic diesel using supported catalysts. *Catal Today* **2008,** 133, 244–254.
